# Comparative pangenomics: analysis of 12 microbial pathogen pangenomes reveals conserved global structures of genetic and functional diversity

**DOI:** 10.1186/s12864-021-08223-8

**Published:** 2022-01-04

**Authors:** Jason C. Hyun, Jonathan M. Monk, Bernhard O. Palsson

**Affiliations:** 1 Bioinformatics and Systems Biology Program, University of California, San Diego, La Jolla, CA USA; 2 Department of Bioengineering, University of California, San Diego, La Jolla, CA USA

**Keywords:** Pangenome, Core genome, Comparative genomics, Multispecies, Heaps’ law, Functional diversity, Sequence diversity, Protein domains, Aminoacyl-tRNA synthetases

## Abstract

**Background:**

With the exponential growth of publicly available genome sequences, pangenome analyses have provided increasingly complete pictures of genetic diversity for many microbial species. However, relatively few studies have scaled beyond single pangenomes to compare global genetic diversity both within and across different species. We present here several methods for “comparative pangenomics” that can be used to contextualize multi-pangenome scale genetic diversity with gene function for multiple species at multiple resolutions: pangenome shape, genes, sequence variants, and positions within variants.

**Results:**

Applied to 12,676 genomes across 12 microbial pathogenic species, we observed several shared resolution-specific patterns of genetic diversity: First, pangenome openness is associated with species’ phylogenetic placement. Second, relationships between gene function and frequency are conserved across species, with core genomes enriched for metabolic and ribosomal genes and accessory genomes for trafficking, secretion, and defense-associated genes. Third, genes in core genomes with the highest sequence diversity are functionally diverse. Finally, certain protein domains are consistently mutation enriched across multiple species, especially among aminoacyl-tRNA synthetases where the extent of a domain’s mutation enrichment is strongly function-dependent.

**Conclusions:**

These results illustrate the value of each resolution at uncovering distinct aspects in the relationship between genetic and functional diversity across multiple species. With the continued growth of the number of sequenced genomes, these methods will reveal additional universal patterns of genetic diversity at the pangenome scale.

**Supplementary Information:**

The online version contains supplementary material available at 10.1186/s12864-021-08223-8.

## Background

With the falling cost of sequencing spurring exponential growth in publicly available genome sequences, genetic analyses have similarly increased in scale over the past three decades, from the first complete microbial genome assemblies in 1995, to comparisons between reference strains of model organisms, and now to simultaneous analyses of thousands of genomes from samples isolated worldwide for multiple species [1, 2]. These pangenome analyses have provided increasingly complete pictures of genetic diversity for most major microbial pathogens, revealing species-level properties invisible at smaller scales, such as the nature of species-wide conserved core genomes compared to their more variable accessory genomes [3, 4], or the tendency for newly sequenced strains of a species to harbor previously unobserved genes, commonly referred to as pangenome openness [5, 6]. Furthermore, pangenomes have formed the basis of many multi-strain characterizations of clinically relevant phenotypes such antimicrobial resistance [7], virulence [8], or metabolic capabilities [9].

However, while the variety of species studied and increasing automation of pangenome workflows [10] attest to the versatility of the pangenome for large-scale genome analysis, pangenome studies are currently dominated by those that focus on one species at a time or combine multiple related species into a single pangenome. Relatively few studies describe methods for comparing distinct pangenomes beyond the sizes of core or accessory genomes: Since Tettelin et.al. introduced the bacterial pangenome and Heaps’ Law as a model for quantifying and comparing openness [6], other multi-pangenome works have compared pangenome openness estimates using alternate models beyond Heaps’ Law [11], level of conservation within core genomes [12, 13], extent of functional characterization in core and pangenomes [14], and functional distributions between core and accessory genomes of different species or environmental isolates [11, 12, 15, 16]. These methods focus primarily on pangenome scaling or the distribution of gene-level functions and are limited in their analysis of finer genetic variation such as individual sequence variants often examined in single pangenome studies. Consequently, existing pangenome studies often present a tradeoff between “scale” (number of species, genomes, or pangenomes analyzed) and “resolution” (smallest unit of genetic diversity analyzed).

To address this gap in pangenome analysis, we present generalizable “comparative pangenomics” methods to examine genetic and functional diversity within and between 12 pangenomes of pathogenic organisms totalling 12,676 genomes. These analyses span several levels of resolution: pangenome shape, individual genes, individual sequence variants, and specific positions within variants. Contextualizing genes against the other three resolutions provides distinct perspectives of diversity at the pangenome scale: 1) gene conservation within the species (core vs. accessory genes), 2) conservation of the gene sequence overall (number and frequency of individual variants), and 3) conservation of specific regions or domains within the gene sequence (positions with high or low diversity within aligned variants). In addition to standard pangenome analyses, we compare functional annotations against these forms of genetic diversity to identify which gene functions are consistently stable against or subjects of major variation across a variety of pathogens.

## Results

### Pangenome construction for reference genome-free enumeration of genetic variation

**Fig. 1:**
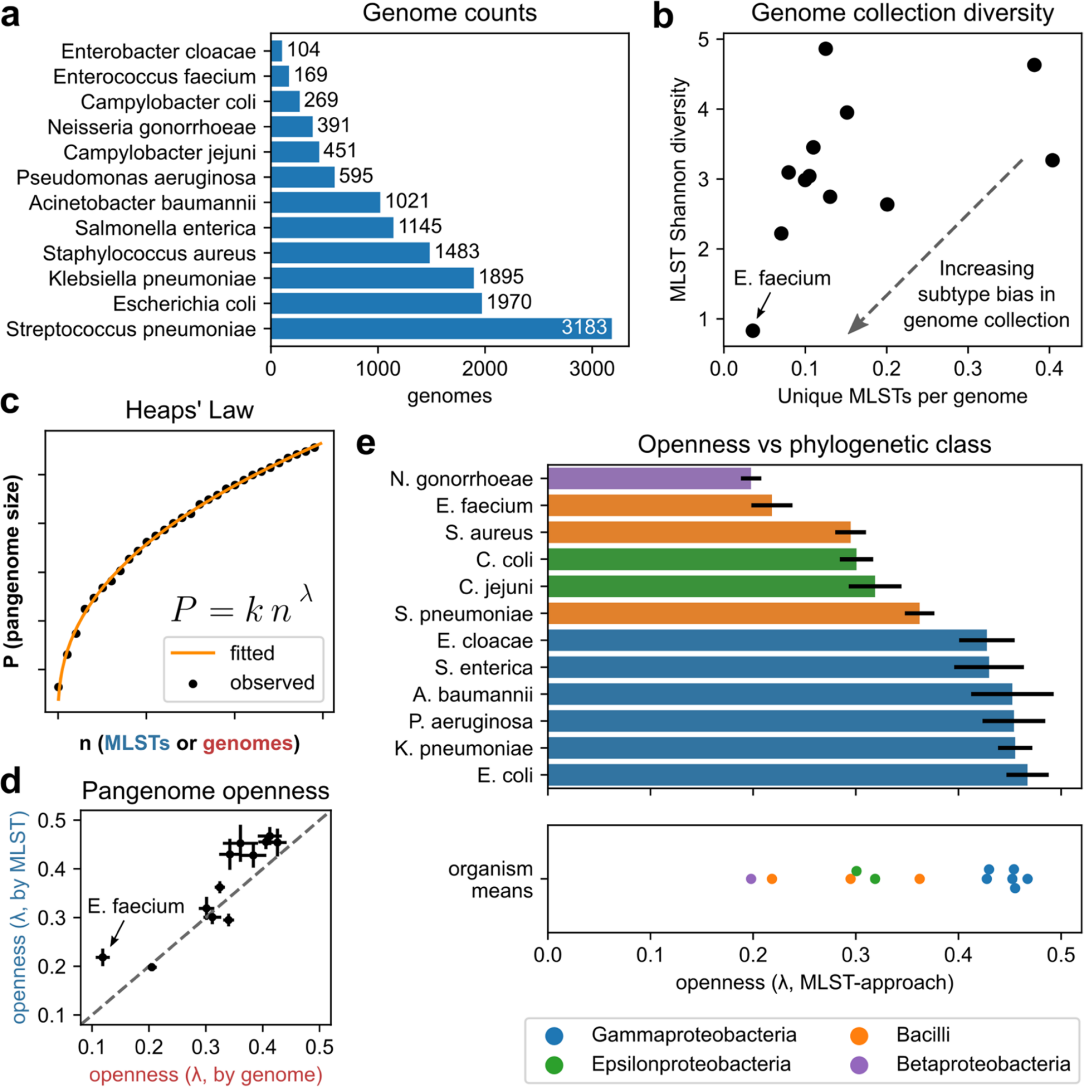
Subtype-balanced Heaps’ Law estimates of pangenome openness for 12 microbial pathogens. **a** Total number of genomes analyzed per species. **b** MLST subtype diversity per species’ genome collection, as quantified by unique MLSTs per genome and Shannon diversity of MLST distributions. Low diversity outlier *E. faecium* is labeled. **c** Example fit of Heaps’ Law between the number of genomes or MLSTs versus running pangenome size. **d** Comparison of openness values estimated per species, with or without controlling for MLST (error bars are standard deviations from 100 estimates). **e** Means and standard deviations of openness estimates after controlling for MLST, versus phylogenetic class

A total of 12,676 genomes across 12 different species were downloaded from the PATRIC database [17] after filtering for assembly quality (see Methods), ranging from 104 to 3183 genomes per species (Fig. 1a, Fig. S1a, Table S1, Dataset S1). Each genome was classified in silico by multilocus sequence type (MLST) (Fig. S1b, Dataset S2) using the mlst tool (https://github.com/tseemann/mlst) based on PubMLST [18]. For each species, a pangenome was constructed by clustering open reading frames by protein coding sequence into putative genes clusters, using CD-HIT [19]. These cluster-derived genes were used to define three other genetic feature types within each pangenome, namely “coding variants” (individual protein sequence variants of the genes, based on members of gene sequence clusters), “5’ IG variants” (DNA variants of the 300nts directly upstream of all observed instances of a given gene), and “3′ IG variants (DNA variants of the 300nts directly downstream of a gene).

### A subtype-based estimate of pangenome size and openness using Heaps’ law is more accurate than genome-based estimates

At the broadest resolution of genetic diversity, pangenome openness, or the tendency for new genomes of a given species to introduce new genes, can be used quantify overall gene-level diversity within a species at the pangenome level as well as project pangenome size as additional genomes are sequenced. Openness is most commonly estimated as the power law exponent when fitting Heaps’ law to pangenome size versus number of genomes, across many iterations of randomly shuffling genome order [6]. This application of Heaps’ Law is based on its original discovery in linguistics as an empirical relationship between the number of unique words encountered and the number of documents reviewed, for which an analogous relationship between genes encountered and genomes sequenced has been observed for multiple bacterial pangenomes [6, 11]. However, MLST classification revealed that the genomes available for some species were highly biased for one or a few subtypes (i.e. 75% of *E. faecium* genomes are from MLST 80), while others were more diverse (Fig. 1b, Fig. S1b). Consequently, estimating pangenome openness based on new genes discovered per genome may in some cases be more characteristic of a single subtype and underestimate overall species-wide openness and/or extrapolate pangenome size poorly.

To address this, we estimated openness with Heaps’ law using two methods to generate 100 random genome orderings per species: 1) the standard approach of randomly shuffling all genomes, and 2) randomly selecting one genome per MLST subtype and shuffling the selected genomes (Fig. 1c). MLST-based estimates of openness were greater than genome-based estimates in 10/12 species without any notable increase in the standard deviation of the estimates, with larger differences observed in more strongly subtype biased cases; the openness estimate for the strongly subtype-biased *E. faecium* case is nearly doubled when using the MLST-based estimate (Fig. 1d, Table S2).

To compare accuracy at extrapolating pangenome size, Heaps’ Law fits were computed on the first half of genomes and evaluated on the second half for all random genome orderings (i.e. for a species of 200 genomes and 20 MLST types, the genome-based approach would be fit to the first 100 genomes and evaluated on the last 100 genomes, while the MLST-based approach would be fit to the first 10 and evaluated on the last 10) (Fig. S2a). The median mean absolute error (MAE) for the MLST-based approach was lower in 11/12 species in the fit region and 9/12 species in the extrapolation region, despite having fewer points to fit (Fig. S2b-c, Table S3). The two cases where the MLST-based approach underperformed the genome-based approach were *P. aeruginosa* (2.0 times larger MAE) and *S. enterica* (1.5 times larger MAE). As the estimated openness and MLST distribution diversity for these species are not particularly different from that of other species, one possible explanation may be due to these cases resulting in relatively poor fits to Heaps’ Law in general, being the 1st and 2nd largest MAE cases by the MLST-based approach and the 3rd and 5th largest MAE cases by the genome-based approach, respectively.

Overall, the MLST-based Heaps’ Law approach appears to extrapolate pangenome size more consistently than a full genome-by-genome approach, and may offer a more accurate depiction of the genetic diversity of a given species even when using subtype-biased datasets. The calculated openness values appear to cluster roughly by species’ phylogenetic classification (Fig. 1e). The top 6 most open pangenomes cluster closely (λ = 0.42-0.47) and consist of the six Gammaproteobacteria class species examined (*E. cloacae, S. enterica, A. baumannii, P. aeruginosa, K. pneumoniae, E. coli*), followed by a group with intermediate openness (λ = 0.29-0.36) of two Bacilli class species (*S. aureus, S. pneumoniae*) and the two Campylobacter species (*C. coli*, *C. jejuni*), and finally the two most closed species (λ = 0.20-0.22) consisting of *E. faecium* (Bacilli) and *N. gonorrhoeae* (Betaproteobacteria).

### Frequency-based division of the pangenome using power functions

**Fig. 2:**
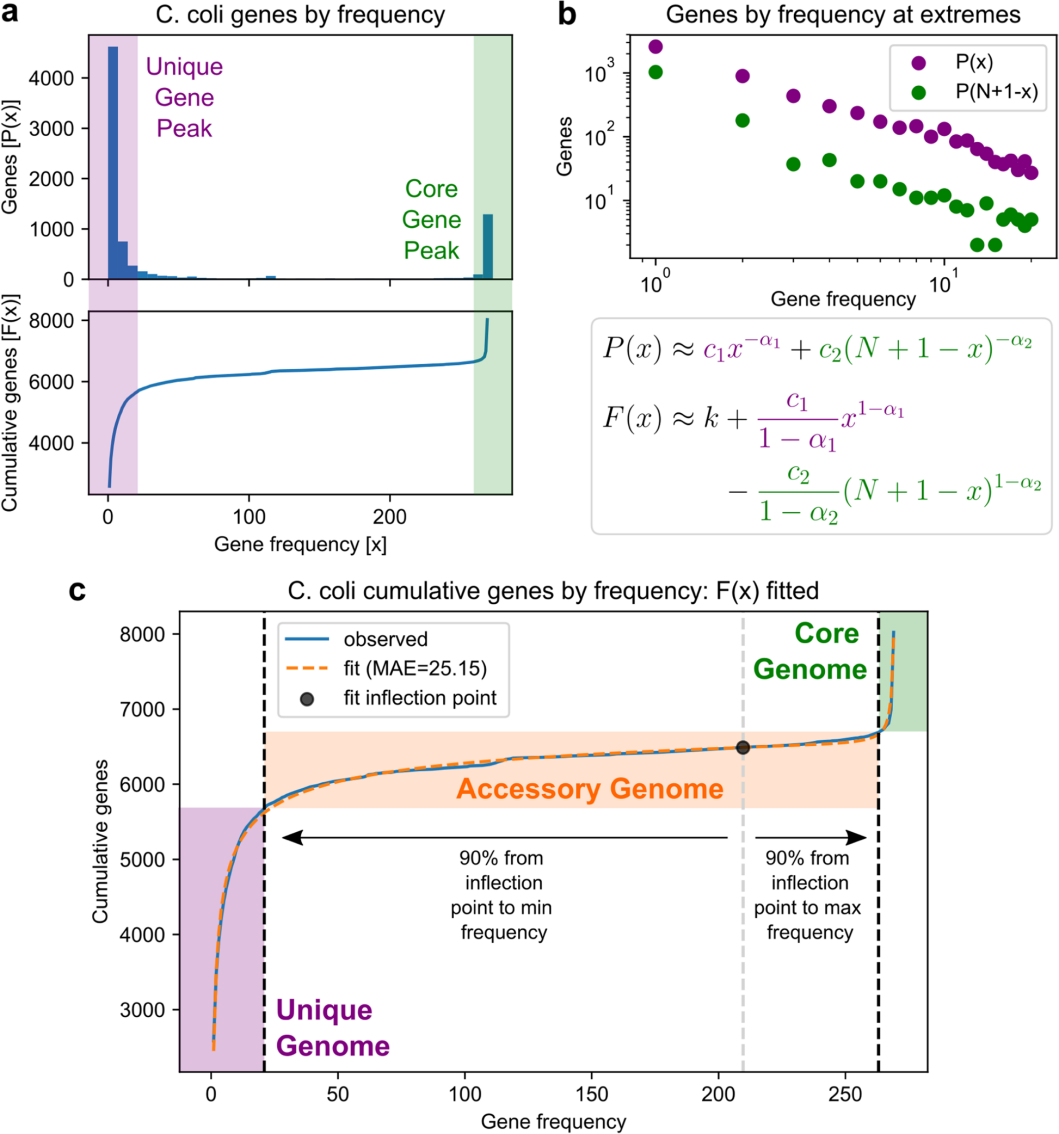
Example division of the *Campylobacter coli* pangenome into unique, accessory, and core genomes. **a** Distribution of gene frequencies P(x), or the number of times a gene is observed in a genome, with peaks at very low (“unique”) and very high (“core”) frequencies. The corresponding cumulative distribution F(x) is shown below. **b** Log-log plots of the frequency distribution at very low and very high frequencies showing approximately linear trends, and the corresponding models of P(x) as the sum of two power functions and F(x) as the integral. N is the total number of genomes. **c** Division of the pangenome into unique, accessory, and core genomes based on the cumulative distribution fit. Frequency thresholds for unique and core genes are defined relative to the fitted inflection point

Moving from the resolution of overall pangenome shape to individual genes, the distribution of gene frequencies (number of genomes each gene is observed in) was computed for each pangenome to begin exploring sources of genetic diversity in greater detail. Regardless of the number of genomes available or the estimated pangenome openness, all such distributions demonstrate a peak for very rare genes and a smaller peak for highly conserved or core genes (Fig. 2a, Fig. S3). Correspondingly, the cumulative gene frequency distribution takes on an asymmetric, inverse sigmoidal shape, which suggests three intuitive frequency categories by which genes may be classified: the initial asymptotic region consisting of rare, poorly characterized genes representing the “unique” genome, the opposite asymptotic region consisting of highly conserved genes representing the “core” genome, and the middle linear region consisting of uncommon genes representing the “accessory” genome which captures most of the gene-level diversity in the pangenome (Fig. 2a).

Three-part frequency divisions of the pangenome have been previously described, and often achieved through either static thresholds such as having core genes being those in all genomes and unique genes being those in exactly one genome [3], or more scalably through fitting frequency distributions to multiple exponential functions to identify analogous “core-shell-cloud” divisions of the pangenome [20, 21]. Here, we developed an approach based on fitting the distribution to the sum of two power functions and defining the accessory genome relative to the inflection point in the cumulative distribution. This functional form is derived from the observation that gene frequency distributions tend to resemble power laws for very small and very large frequencies (Fig. 2b-c), and achieves accurate fits to cumulative frequency distributions with MAE ranging from 25 to 116 genes, less than 0.5% of the total pangenome size for 11/12 species. The fits also achieve R^2^ > 0.99 for 10 of 12 pangenomes and a minimum of 0.964 (Fig. S4, Dataset S3). The frequency thresholds for core-accessory-unique divisions defined from these fits ranged from 95.8 to 98.6% of all genomes for core genes, and 5.8 to 8.6% for unique genes, highlighting the asymmetry present in the original frequency distributions (Table S4).

**Fig. 3:**
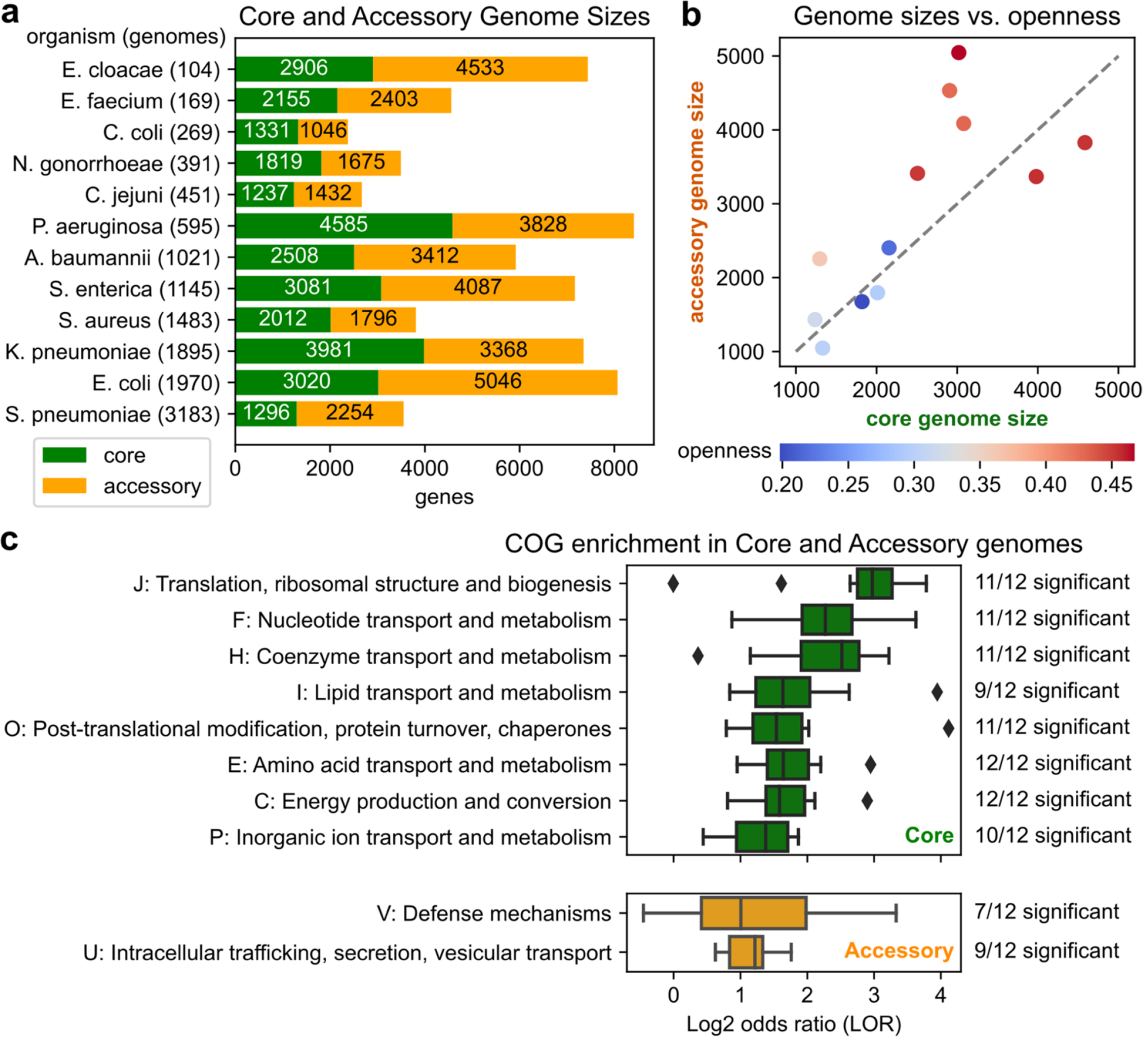
Genes and functional enrichments in the core and accessory genomes of 12 species. **a** Number of genes in the core and accessory genomes of each species. **b** Comparison of core genome size, accessory genome size, and pangenome openness. **c** Functional enrichments by COG functional category in the 12 core and accessory genomes. The distribution of log2 odds ratios (LORs), as well as the number of species with significant enrichment by COG are shown (Fisher’s exact test, FWER < 0.05 under Bonferroni correction or *p* < 7*10^− 5^, 720 tests). Only COGs showing positive enrichment in over half the species and with mean LOR > 1 are shown. COG “S: Function unknown” is not shown

This pangenome division approach yielded core genomes ranging from 4.3 to 34.2% of their corresponding pangenomes with a minimum of 1237 core genes in *C. jejuni* to a maximum of 4585 in *P. aeruginosa*, while accessory genomes ranged from 5.0 to 38.1% of their corresponding pangenomes with a minimum of 1046 accessory genes in *C. coli* to 5046 in *E. coli* (Fig. 3a, Table S4). Core genomes were similar in size to corresponding accessory genomes and larger genomes were associated with more open pangenomes, though there was no relationship between the ratio of core to accessory genome size and openness (Fig. 3b). Overall, by creating three frequency categories, this method allows subsequent analyses to focus on a smaller number of genes (relative to full pangenomes) such as highly conserved core genes or accessory genes that constitute most of the gene-level diversity in an species, rather than the more abundant but often under-characterized or erroneous unique genes.

### Consistent enrichment of specific gene functions in core and accessory genomes

To identify associations between gene frequency and function, all genes were annotated for Clusters of Orthologous Groups (COG) functional categories and GO terms using eggNOG-mapper [22], and Fisher’s exact tests were conducted between each frequency category and COG category within each pangenome (Dataset S4). This revealed consistent enrichment of several metabolic COGs in the core genome, with COGs C (energy production and conversion), E (amino acid transport and metabolism), F (nucleotide transport and metabolism), and H (coenzyme transport and metabolism), as well as non-metabolic COGs J (translation, ribosomal structure and biogenesis) and O (post-translational modification, protein turnover, and chaperones), significantly enriched in the core genome for at least 11/12 species (*p* < 7*10^− 5^, FWER < 0.05 with Bonferroni correction) and mean log2 odds ratios (LOR) ranging from 1.7 to 2.8 across the species (Fig. 3c, Fig. S5a). Accessory genomes also showed frequent, albeit weaker functional enrichments, with two COGs with mean LOR > 1 across the species: U (intracellular trafficking, secretion, and vesicular transport) was enriched in 9/12 species with a mean LOR of 1.2, and V (defense mechanisms) enriched in 7/12 species also with a mean LOR of 1.2 (Fig. 3c, Fig. S5b). Finally, with the exception of COG S (function unknown), no COGs were found with either frequent significant enrichment or mean LOR > 1 in the unique genomes, owing to their relatively poor characterization (Fig. S5c).

A similar analysis of GO terms revealed that 9 of the top 10 enriched GO terms in core genomes by mean LOR were associated with ribosomes or RNA processing (LOR = 3.8-6.9). All but one of those terms was also significantly enriched in at least 11/12 species (*p* < 3*10^− 6^, FWER < 0.05 with Bonferroni correction), consistent with the J COG previously found enriched in core genomes (Fig. S6a). In contrast, no GO terms were found to be significantly enriched in a majority of accessory or unique genomes, with the exception of very broad terms such as “cellular process” (Fig. S6b-c). Overall, this functional analysis suggests that the core genomes of microbial pathogens are likely enriched for metabolic and translational functions, while non-core genes may draw from a wider variety of relatively niche functions.

**Fig. 4:**
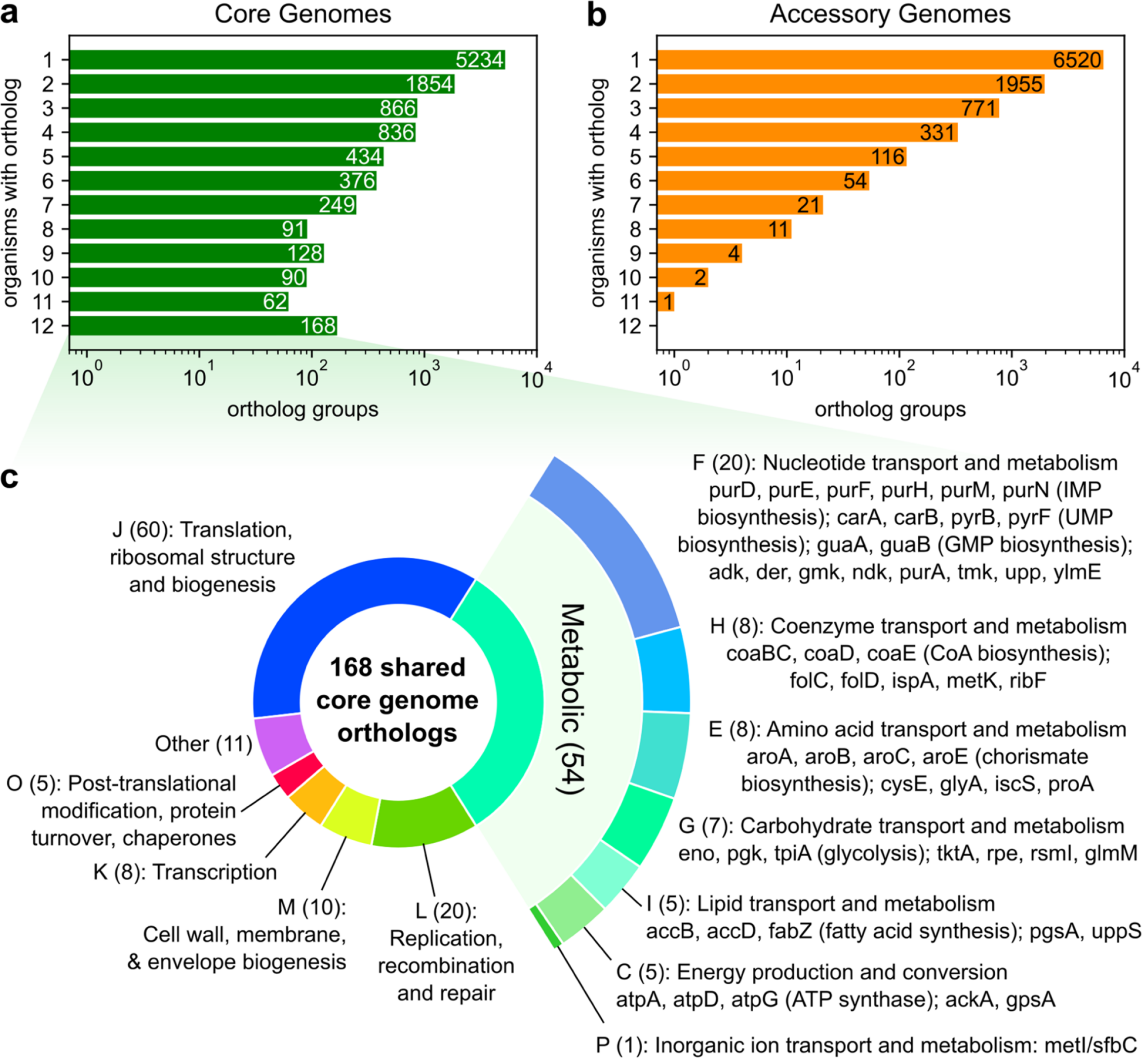
Distribution of shared genes in core and accessory genomes. Number of shared genes versus frequency of observation across the **a** core genomes and **b** accessory genomes of 12 species. **c** Functional breakdown of the 168 genes observed in all 12 core genomes. Colors correspond to individual COG functional categories, which are labeled by the number of shared core genes annotated with the COG and COG definition. For metabolic COGs, individual genes and associated pathways are listed

Finally, an examination of individual ortholog groups (OGs) as annotated by eggNOG-mapper reveals specific biosynthetic pathways consistently present in core genomes. 168 OGs were found in all 12 core genomes (Fig. 4a), while the most common OG among accessory genomes was found in 11 accessory genomes (Fig. 4b). A majority of these conserved core OGs were found to be essential for growth for *E. coli* in LB media (101/168, 60%) [23] (Dataset S5). Functionally, core OGs were again dominated by translation/ribosomal genes (60/168, 36%) and also included a significant number of metabolic genes (54/168, 32%), many of which share metabolic pathways (Fig. 4c). Purine metabolism was strongly represented with 15 OGs conserved in all core genomes: *purD, purE, purF, purH, purM, purN* in IMP biosynthesis; *carA, carB, pyrB, pyrF* in UMP biosynthesis; *guaA, guaB* in GMP biosynthesis*; purA* in AMP biosynthesis; and *adk*, *gmk*, *upp* in salvage pathways. Other conserved OGs sharing biosynthetic pathways include *aroA, aroB, aroC, aroE* in chorismate biosynthesis; *coaBC, coaD, coaE*, in coenzyme A biosynthesis, and *accB, accD, fabZ* in fatty acid biosynthesis.

### Genes conserved at the sequence level are enriched for translation-associated genes, while sources of core genome sequence diversity are functionally diverse

**Fig. 5:**
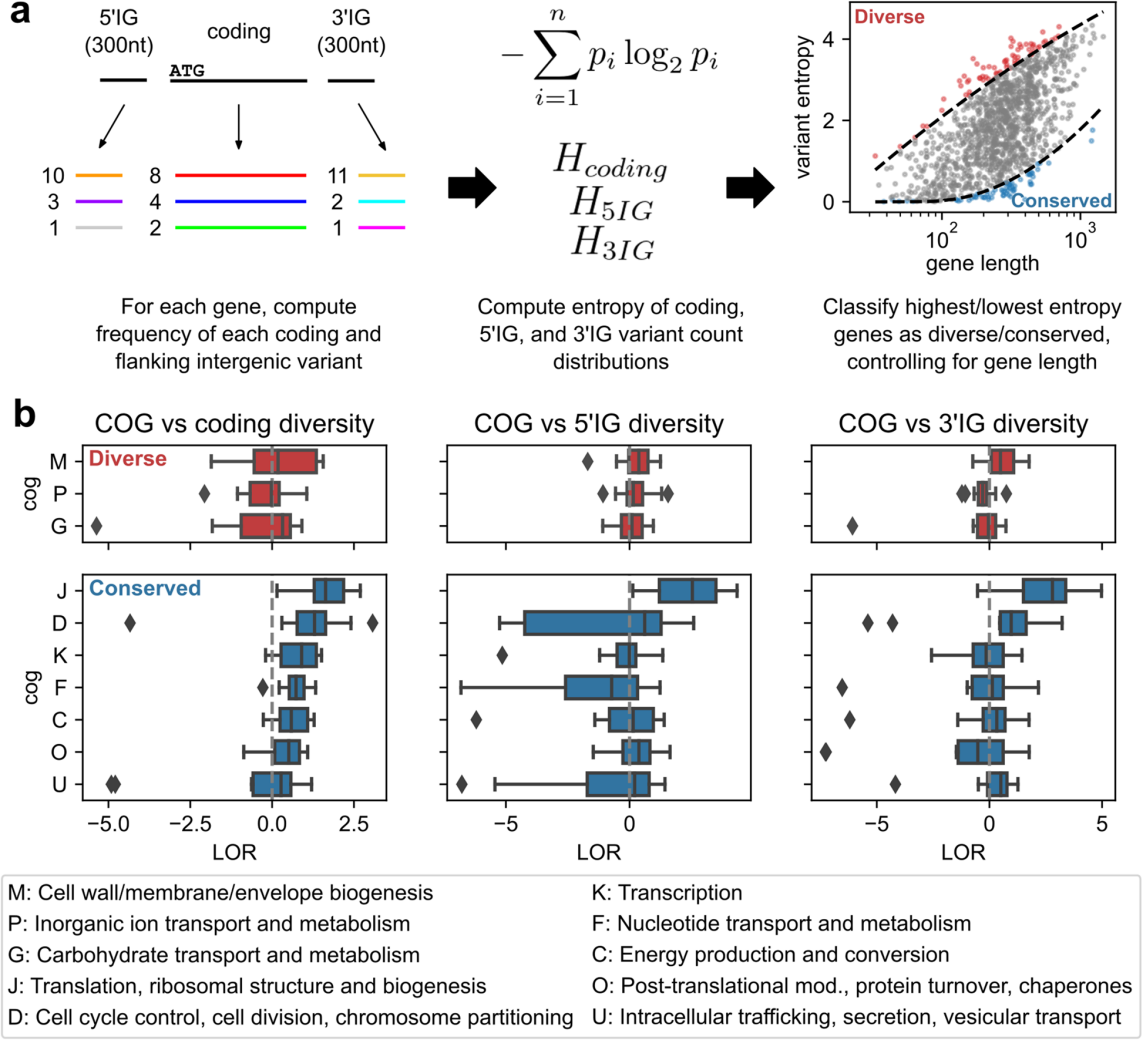
Functional enrichment in core genes versus sequence diversity in coding or flanking intergenic sequences. **a** Workflow for identifying genes with high or low sequence diversity. For a given gene and species, frequencies of individual coding, 3′ intergenic (3’IG), and 5′ intergenic (5’IG) variants were computed, and entropies of the three variant type-specific frequency distributions were computed as measures of sequence diversity. For a given entropy measure, genes in the top and bottom 5% were classified as “diverse” or “conserved”; in the case of coding sequence entropy, 5 and 95% percentiles as a function of gene length were used instead, estimated through quantile regression. **b** Functional enrichment in genes classified as most diverse or conserved by either coding, 5’IG, or, 3’IG sequence entropy. Only COGs with positive mean log2 odds ratio (LOR) across the 12 species for at least one entropy measure are shown

Moving to the resolution of individual variants to assess sequence-level genetic diversity, the frequency of each unique protein sequence associated with each core gene within each species’ pangenome was computed. The entropies of these variant frequency distributions were computed as a measure of coding sequence diversity for each gene. Similarly, the entropies of analogous frequency distributions for gene-specific 5’IG and 3’IG variants were also computed, resulting in three “allelic entropy” measures for each gene (Fig. 5a, see Methods for entropy calculations). These measures allow for the quantification of a gene’s overall sequence-level diversity, without requiring reference genomes or computationally expensive multiple sequence alignments for each sequence cluster that would be infeasible at the multi-pangenome scale. For each species’ core genome, only limited correlation was observed between the level of sequence variability in a gene’s coding sequence compared to flanking intergenic sequences; median Spearman correlation across the 12 species was 0.286 between coding and 5′ upstream allelic entropy, and 0.237 between the coding and 3′ downstream allelic entropy (Table S5).

To identify the most and least sequence diverse core genes by each feature type (coding, 5’IG, or 3’IG), the top and bottom 5% of core genes were identified after sorting by the corresponding allelic entropy measure and classified as “diverse” or “conserved”, respectively. In the case of coding sequence diversity, since the metric is sensitive to gene length, the top and bottom 5% of genes were instead identified using quantile regression [24] to estimate the 5 and 95% allelic entropy percentiles as a function of gene length (Fig. S7, Fig. S8). Functional enrichment tests between COG functional groups and either the most or least conserved core genes per species revealed generally little association between any of the types of sequence diversity and gene function (Fig. 5b). Only COG J (translation, ribosomal structure and biogenesis) exhibited consistent enrichment among the least sequence-diverse core genes, with mean LORs across the 12 species of 1.7, 2.4, and 2.4 among genes conserved by coding, 5’IG, and 3’IG allelic entropy, respectively, and statistically significant enrichment in 7/12 species for all three measures (*p* < 7*10^− 5^, FWER < 0.05 with Bonferroni correction). Additional weak biases towards other COGs were also observed for conserved or diverse genes by one or more feature types, though none were statistically significant in more than single species (Fig. 5b, Dataset S4).

Finally, genes involved in PubMLST typing schemes for these species ranged from strongly conserved to highly diverse based on coding allelic entropy (Fig. S9): all MLST genes were classified as core genes, and the most coding diverse gene in each scheme ranged from the 72nd to 99th percentile in the coding allelic entropy distribution of the corresponding species’ core genome, while the least coding diverse gene ranged from the 4th to 36th percentile.

### Position of variation in conserved core genes is domain-dependent, especially among aminoacyl-tRNA synthetases

**Fig. 6:**
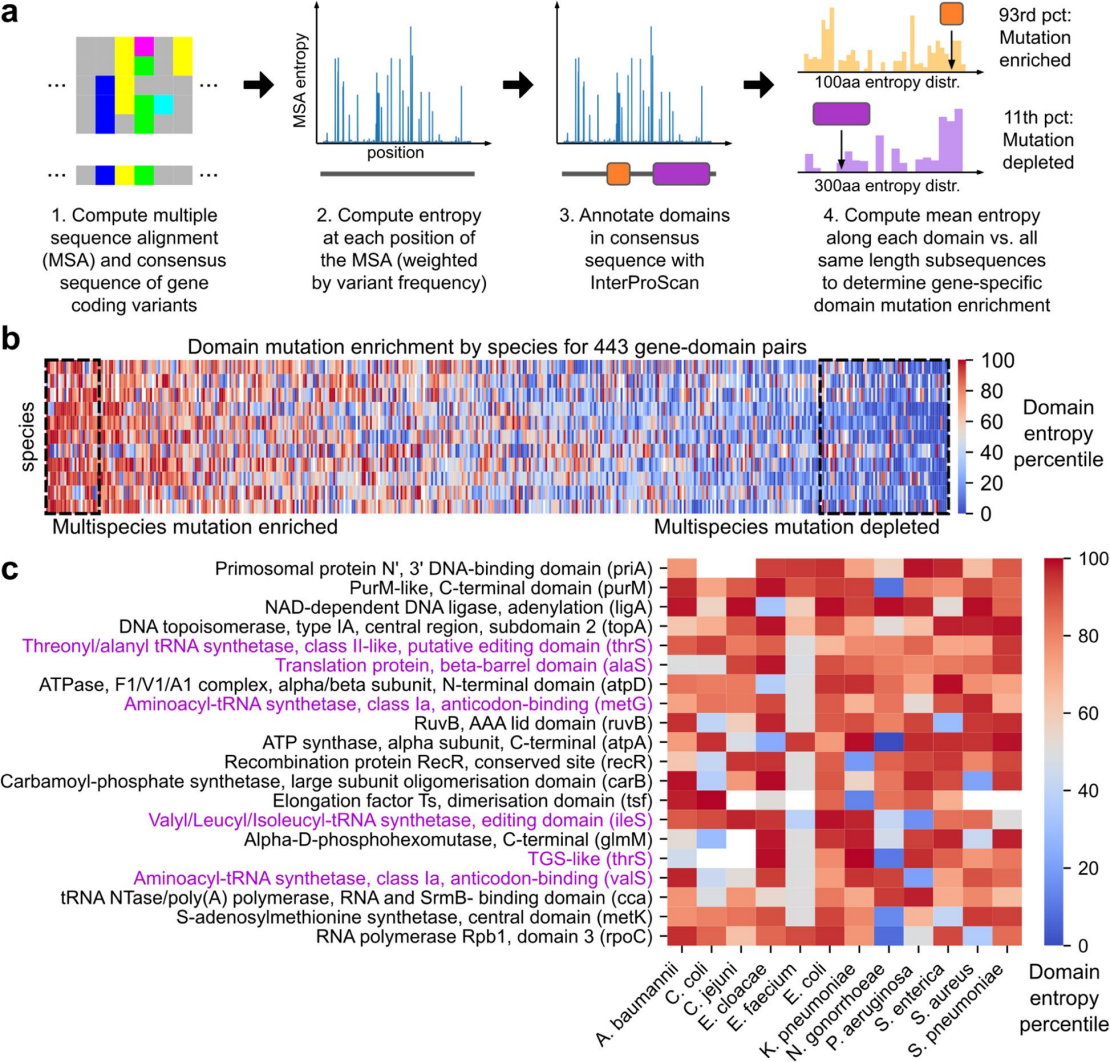
Mutation enrichment in protein domains from 76 genes present in all 12 species’ core genomes. **a** Workflow for computing the extent of mutation enrichment in a domain relative to the full protein from a set of coding variants. Briefly, the entropy at each position of the gene’s multiple sequence alignment is computed, and the mean entropy across the length of the domain is compared to that of all same length subsequences of the protein to compute a domain entropy percentile. **b** Species-specific mutation enrichment for 443 gene-domain pairs, sorted by domain entropy percentile averaged across 12 species. Domains with statistically significant multispecies enrichment or depletion are boxed (Bootstrap test, FDR < 0.05, Benjamini-Hochberg correction). *E. faecium* is not shown, due to low variability attributable to initial subtype imbalances in the genome set. **c** Species-specific mutation enrichment for gene-domain pairs with significant multispecies mutation enrichment. Domains related to aminoacyl-tRNA synthetases are labeled purple. White cells correspond to domains that could not be annotated within the species’ consensus sequence for the parent protein

Finally, for the highest resolution of pangenomic diversity, the position of sequence-level diversity in the pangenome was examined for 76 of the 168 OGs (here on referred to as just “genes”) previously found to be in all 12 core genomes after filtering for those that could be richly annotated for domains (see Methods for gene selection process). For each species-specific set of protein sequence variants of a given gene, a multiple sequence alignment (MSA) was computed using MAFFT [25], from which the consensus sequence was annotated for domains using InterProScan [26]. The entropy at each position of the MSA was computed, and to evaluate a domain’s variability relative to its parent gene, the mean MSA entropy across all positions spanned by the domain was computed and compared against the mean MSA entropies of all windows with the same length as the domain in the MSA, yielding the domain’s entropy percentile with respect to the given gene and species (Fig. 6a).

**Fig. 7:**
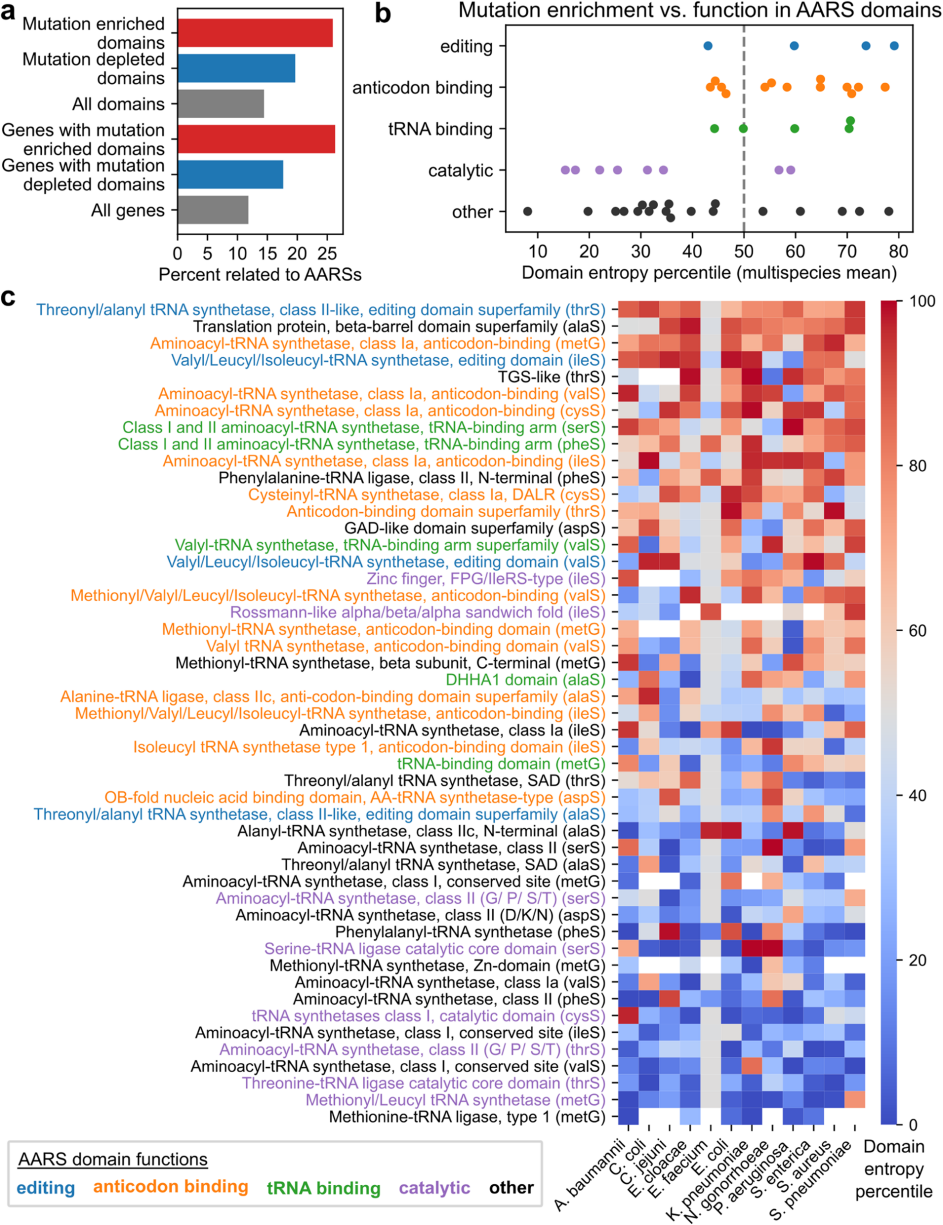
Species-specific mutation enrichment among aminoacyl-tRNA synthetase domains relative to corresponding full proteins. **a** Enrichment of aminoacyl-tRNA synthetase (AARS) related features among all domains with significant mutation enrichment or depletion. **b** Extent of mutation enrichment in AARS domains compared to function across 12 species. Each point corresponds to a single gene-domain pair, categorized by function based on InterPro descriptions. **c** Species-specific mutation enrichment for all AARS-associated gene-domain pairs, sorted by domain entropy percentile averaged across the 12 species. White cells correspond to domains that could not be annotated within the species’ consensus sequence of the parent protein

Across the 443 gene-domain pairs analyzed, 27 domains were identified to be mutation enriched with significantly elevated entropy consistently across the 12 species analyzed, and 61 domains were identified to be mutation depleted with significantly reduced entropy (Bootstrap test, FDR < 0.05, Benjamini-Hochberg correction) (Fig. 6b, Dataset S6). Both the mutation enriched and mutation depleted domains are functionally diverse and are found in a wide range of genes (Fig. 6c, Fig. S10), though with a bias towards domains related to aminoacyl-tRNA synthetases (AARSs); 26% of mutation enriched domains were related to AARS compared to 14% of the full set of domains analyzed (Fig. 7a). A survey of AARS-related domains finds that the extent of a domain’s multispecies mutation enrichment is associated with function (Fig. 7b-c). Among the 9 AARSs analyzed (*alaS, aspS, cysS, ilesS, metG, pheS, serS, thrS, valS*), domains related to editing, anticodon binding, or tRNA binding were either mutation enriched or mutation neutral on average across the 12 species, while all but two non-editing catalytic domains were mutation depleted. Other domains (structural and/or of unknown function) were distributed over the full range from mutation depletion to enrichment.

Within individual AARS domain functional categories, variability in mutation enrichment was due primarily to gene differences (i.e. differences in catalytic domains between *metG* vs. *ileS*) and lesser so to annotation specificity (i.e. domain vs. superfamily annotation of a similar region) (Fig. 7c). Outliers due to gene include the FPG/IleRS-type Zinc finger domain and Rossman-like alpha/beta/alpha sandwich fold domain in *ileS*, the only catalytic domains to be mildly mutation enriched (compared to the catalytic domains of *serS*, *cysS*, *thrS*, and *metG* which are strongly mutation depleted); and the putative editing domain of *alaS*, which is mildly mutation depleted compared to the strongly mutation enriched editing domains of *thrS*, *ileS*, and *valS*.

## Discussion

Advances in sequencing technologies have rapidly expanded the scale of public genome collections, allowing the scope of analyses to grow from full genomes, to multiple genomes, and now towards multiple pangenomes for global comparisons of genetic diversity between species. However, though numerous studies have examined genetic and functional diversity present in individual pangenomes, relatively few have offered comparisons between multiple distinct pangenomes, especially at the resolution that single pangenome studies often explore. In this study, we present generalizable “comparative pangenomics” methods for contextualizing genetic diversity with function across multiple species and at multiple resolutions, from the shape of the pangenome overall to specific positions within individual genes.

At the overall pangenome level, we find that by balancing representation of MLST subtypes through undersampling, Heaps’ Law more accurately predicts pangenome size as new genomes are introduced while yielding pangenome openness estimates with variances similar to that of estimates derived without balancing. The balanced openness estimates were frequently higher than those derived without balancing (9/12 cases), possibly due to the estimates being less biased towards any individual subtype which typically draws from a more limited gene pool than the species overall. Such a trend was observed in clade-specific analyses of the *E. coli* ST131 pangenome, where all but one clade was more closed than the combined population [27]. Furthermore, the estimates suggest that openness roughly follows phylogenetic placement, especially with all six Gammaproteobacteria species analyzed here having very similar openness values that are all higher than that of the other bacterial classes examined. This is mostly corroborated in previous comparative works, though the exact openness values differ from those calculated here. Park et.al. found four Gammaproteobacteria, *A. baumannii*, *E. coli*, *S. enterica*, and *P. aeruginosa*, to have similar openness values compared to three other species analyzed from different phylogenetic classes [11], and Tettelin et.al. classified *E. coli* and *S. pneumoniae* as open and *S. aureus* as relatively closed [6]. It is possible that subtype balancing is responsible for the differences in exact openness values, and ultimately the results suggest that integrating subtype information into models of pangenome size may more accurately reflect the level of genetic diversity within the species at this scale.

Moving from overall pangenome shape to individual genes, an examination of gene frequency distributions reveals that a double power function can closely model such distributions (*R*^*2*^ > 99% in 11/12 species) and provides a scalable method for dividing the pangenome into frequency categories core, accessory, and unique. This approach is similar to the core-shell-cloud division based on a triple exponential function described by Koonin and Wolf [20] and implemented in the GET_HOMOLOGUES pangenome pipeline [21], which was similarly derived based on examining functional forms that closely fit empirical distributions, albeit originally for smaller genome collections. Future analyses may examine which functional form offers closer and more stable fits at scales of thousands of genomes, and how they compare to more sophisticated approaches generalizable to more than three partitions, such as PPanGGOLiN’s integration of both gene frequency and synteny conservation information [28], or micropan’s use of binomial mixture models [29].

An analysis of gene function distributions across these frequency categories finds several functional categories to be consistently associated with frequency across most of the examined species. Translation/ribosomal genes, as well as a number of genes from specific metabolic categories were significantly enriched in nearly all core genomes examined, while those concerning more niche functions such as trafficking/secretion or defense mechanisms were significantly enriched in a majority of accessory genomes. These results are also partially corroborated in Park et.al., where translation genes were among the top 5 overrepresented functional categories in 3/7 core genomes, trafficking/secretion in 2/7 accessory genomes, and various metabolic categories also overrepresented in some core genomes [11]; differences may be attributed to a more restrictive reporting (only top 5 categories are shown rather than all statistically significant cases), as well as a different statistical setup resulting in the reporting of some categories (such as transcription- or replication-associated) as overrepresented in both the core and accessory genomes. Additionally, the enrichments found here, especially that of translation genes in core genomes, were recovered in more focused studies examining 1-3 species or genuses at a time, such as for *A. baumannii* [30], Campylobacter [31], *E. coli* [32], *E. faecium* [33], *N. gonorrhoeae* [34], *P. aeruginosa* [35, 36], and *S. aureus* [37]. Finally, an analysis of individual genes identified 168 genes in the core genome of all 12 species, which were predominantly genes essential in *E. coli* (60%) and follow a functional distribution similar to that of core genomes overall, composed primarily of translation (36%) and metabolic (32%) genes (especially in nucleotide metabolism). This functional breakdown strongly resembles that of the “minimal gene set” identified in 1996 by Mushegian and Koonin for three species in one of the earliest characterizations of a bacteria-wide conserved gene set [38]. The repeated observation of specific functional enrichments in both this work and others suggest that core and accessory genomes from a wide array of bacterial species may share a consistent structure regarding functional distribution.

At the level of individual variants, we find less consistency within and between species regarding sequence-level genetic diversity. Using entropy of variant distributions to quantify sequence-level diversity without reference genomes or computationally expensive multiple sequence alignments, we find that the level of variability within a core gene’s coding sequence is only weakly correlated with that of its immediate 5′ or 3′ flanking intergenic region in all 12 species examined (Spearman correlation between 0.2-0.3). Pangenome-wide disparities in variation between the coding and flanking intergenic regions of a gene have been previously observed at the gene level: at least 11% of *E. coli* core genes were found to exhibit “regulatory switching” between nonhomologous flanking intergenic regions [39], and 7% of *S. aureus* core genes were found adjacent to non-core intergenic sequences [40]. Furthermore, while translation/ribosomal genes were consistently overrepresented among the genes most strongly conserved at the sequence-level, the functional distribution of core genes responsible for the most sequence-level variability differs significantly by species. Whereas the functional distribution of overall gene content may be relatively stable between species, this finer-grained, shorter-term genetic diversity appears to impact a much broader range of functions within and across different species.

At the highest resolution assessment of genetic diversity, applying multiple sequence alignment and domain annotation to shared core genes revealed that specific structural features are disproportionately more conserved or diverse than the remainder of their parent gene, consistently across multiple species. Domains from AARS genes especially tended to exhibit this tendency for multispecies mutation depletion or enrichment, and an AARS-specific analysis revealed that the level of mutation enrichment strongly followed domain function, with non-editing catalytic domains being consistently mutation depleted, while tRNA-binding, anticodon-binding, and editing domains tending to be mutation enriched. This finding of short-term, intraspecies divergence of AARSs being localized away from catalytic domains for multiple species is consistent with previous analyses examining longer-term, interspecies differences in AARSs. Comparisons between representative AARSs of different species have shown significant diversity in overall domain architecture between different species and AARS classes in general [41], but catalytic domains are observed to be most frequently conserved at this level [42].

Additionally, two exceptions were observed in the broader trends between AARS domain function and mutation enrichment. First, the catalytic domains in *ileS* were the only catalytic domains not to be mutation depleted. One potential explanation may be that mutations near the catalytic Rossman fold of *ileS* have been associated with mupirocin resistance in *S. aureus* [43], and we find the Rossman fold domain of *ileS* to be more mutation enriched in *S. pneumoniae* and *S. aureus* compared to naturally mupirocin-resistant *P. aeruginosa* [44]. Second, the editing domain of *alaS* is the only editing domain not to be mutation enriched, while the editing domains of *thrS* and *ileS* are among the most significantly mutation enriched across all domains examined. This result may be interpreted as possible instances of amino acid-specific misaminoacylation being tolerated or even improving fitness under certain stressful conditions, as previously observed for specific amino acids and environments [45, 46]. For example, editing-deficient *ileS* increases the growth rate of *E. coli* under isoleucine starvation [47] and the loss of *thrS* editing may trigger responses against oxidative stress [48], while the loss of *alaS* fidelity is poorly tolerated in *E. coli* [49]. Altogether, this domain analysis offers a finer-grain contextualization of pangenome-scale genetic diversity, revealing broadly conserved patterns of how mutations are localized in conserved genes as well as exceptions that may be explained by specific environmental stresses.

Finally, we note that pangenome-scale analyses are always limited by the availability of high quality genome assemblies and will continue to improve as more sequences are published. Future development and application of these methods to larger genome collections will provide increasingly complete pictures regarding the full extent of genetic diversity within a species, as well as present new challenges in evaluating the completeness and evenness of represented subtypes. Furthermore, similar analyses of additional species are necessary to determine whether the patterns of genetic diversity observed here are also present more broadly across the bacterial domain beyond major human pathogens.

## Conclusions

Overall, in developing efficient and generalizable methods for pangenome analysis, we find that each resolution of the pangenome reveals distinct aspects of the relationship between genetic and functional diversity across multiple species located across the phylogenetic tree. In increasing resolution, we find across 12 pathogenic species that pangenome openness is associated with phylogenetic placement, the distribution of gene functions in the core and accessory genome is conserved across species, short-term sequence variation in core genomes impacts a functionally diverse range of genes, and certain protein domains are enriched for mutations consistently across multiple species in a function-dependent manner, especially among AARSs. Many of the conserved patterns of genetic diversity uncovered here are consistent with previous studies focused on individual species, and continued development of multi-scale comparative pangenomic techniques may further elucidate similarities in how different species adapt to their environmental niches and pressures.

## Methods

### Genome selection, pangenome construction, MLST classification, and feature identification

An initial set of genomes was taken from the PATRIC database RELEASE_NOTES (ftp.patricbrc.org/RELEASE_NOTES/, 2020-02-06), starting with ESKAPEE pathogens and WHO global priority pathogens and filtered down to 12 species with at least 100 genomes by taxon ID (Table S1). For each species, genomes were filtered to those meeting the following quality criteria: 1) genome status is “WGS” or “Complete”, 2) number of contigs is within 2.5 times the median number of contigs across all assemblies for that species, 3) number of annotated CDSs is within 3 standard deviations of the mean, and 4) total genome length is within 3 standard deviations of the mean. PATRIC Genome IDs for the selected genomes are available in Dataset S1. Each genome was classified in silico by multilocus sequence type (MLST) using the mlst tool v2.18.0 (https://github.com/tseemann/mlst) based on PubMLST [18] (Dataset S2). A phylogenetic tree was constructed based on reference genomes of each species available on PATRIC, using PATRIC’s Phylogenetic Tree service with the Codon Trees method and a maximum of 100 genes (Fig. S1a) [50]. In the cases of *C. coli* and *A. baumannii*, no reference genome was available on PATRIC and representative genomes were used.

For a given species, all CDSs across all genomes (as annotated by PATRIC) were reduced to a non-redundant list and clustered by protein sequence using CD-HIT v4.6 (word size “-n” 5, minimum identity “-T” 80%, minimum alignment length “-aL” 80%, all other settings default) [19]. Each cluster was denoted a “gene” and each cluster member denoted a coding variant. For each gene, 5′ intergenic variants were identified by locating occurrences of all coding variants of the gene across all genome assemblies and extracting the DNA sequence from the start codon to 300 nt upstream. 3′ intergenic variants were similarly identified downstream of stop codons. Intergenic variants truncated by contig boundaries were ignored.

### Pangenome openness estimation and size extrapolation with heaps’ law

To estimate pangenome openness for a given species, 100 random genome orderings were generated using two approaches: 1) *genome-based*: all available genomes were randomly shuffled, and 2) *MLST-based*: all identified MLST types were randomly shuffled and one genome was randomly sampled per MLST in the resulting order (genomes that could not be typed were grouped as a single separate subtype). For each genome ordering, the total number of unique genes encountered (pangenome size) as genomes are introduced sequentially was computed and fit to Heaps’ Law using nonlinear least squares regression via SciPy’s scipy.optimize.curve_fit() [51]. The mean and standard deviation of fitted Heaps’ Law parameters across the 100 orderings for each method were computed.

To evaluate each method’s ability to extrapolate pangenome size, Heaps’ Law was fit to the first half of genomes in each genome ordering, and the mean absolute error (MAE) was computed for the fit against both the first half (fit region) and remaining second half (extrapolation region) of genomes. The median MAE across the 100 orderings was computed for both methods for each species, as well as the relative median MAE (median MAE from the MLST-based approach divided by the median MAE from the genome-based approach).

### Frequency-based division of pangenomes into core, accessory and unique genes

For a given species with N genomes, two distributions were computed: P(x), the number of genes with frequency x, and F(x), the cumulative genes with frequency less than or equal to x. To account for the observation that P(x) and P(N + 1-x) are approximately power laws for small values, the overall frequency distribution was modeled using the following function with parameters (c_1_,c_2_,a_1_,a_2_):

$$P\left(x\right)=c_1x^{-\alpha_1}+c_2\left(N+1-x\right)^{-\alpha_2}\hspace{3em}x=1,2,\dots,N$$

Since observed P(x) values varied across multiple orders of magnitude, parameters of this function were fitted using the cumulative distribution, based on the integral of the P(x) model and involving an extra constant parameter k:

$$F\left(x\right)=k+\frac{c_1}{1-\alpha_1}x^{1-\alpha_1}-\frac{c_2}{1-\alpha_2}\left(N+1-x\right)^{1-\alpha_2}$$

This five parameter function was fit to observed cumulative frequency distributions, using nonlinear least squares regression via SciPy’s scipy.optimize.curve_fit() [51], linearly scaling the domain and range to be within 0-1 and with initial guess (c_1_,c_2_,α_1_,α_2_,k) = (1,1,2,2,1). The inflection point of F(x), or x*, was computed by minimizing P(x) with the corresponding computed parameters in SciPy using scipy.optimize.minimize_scalar() [51]. Frequency thresholds for core, accessory, and unique genes were defined relative to this inflection point, where unique genes were defined as those present in less than 0.1x* strains, core genes as those present in more than 0.9 N + 0.1x* strains, and accessory genes as everything in between. Fitted parameters and derived frequency thresholds are available in Dataset S3.

### Ortholog group identification and enrichment testing between gene function and frequency

For each gene in each pangenome, the most commonly observed coding variant was annotated using eggNOG-mapper version 0.12.7 [22], as the representative for that gene. This annotation yielded for each gene its best ortholog group or “bestOG”, COG functional category, and associated GO terms. Genes that eggNOG-mapper failed to annotate were assigned the COG category “S: Function unknown”. For each species, Fisher’s exact tests were applied to determine enrichment between each gene frequency group (core, accessory, unique) and COG functional category. For example, to test enrichment for COG J in the core genome, Fisher’s exact test was applied between core vs. non-core genes and COG J vs. non-COG J genes. A total of 12 species × 3 frequency groups × 20 COGs = 720 tests were conducted, and significance was determined based on FWER < 0.05 under Bonferroni correction, or *p*-value < 7*10^− 5^. Log2 odds ratios (LOR) were also computed between each frequency group and COG.

Analogous enrichment tests and LOR calculations were conducted for 414 GO terms that were observed at least 10 times in each species. A total of 12 species × 3 frequency groups × 414 GO terms = 14,904 total tests were conducted, and significance was determined based on FWER < 0.05 under Bonferroni correction, or p-value < 3*10^− 6^. The top 10 GO terms by mean LOR across all 12 species were reported. All LORs and *p*-values for both COG and GO terms are available in Dataset S4.

To identify genes conserved across all species’ core genomes, genes from different species’ pangenomes were grouped by eggNOG-mapper’s bestOG assignment. Gene essentiality was assigned based on comparing eggNOG-mapper predicted gene names to essentiality predictions made in Goodall et.al [23]. and are available in Dataset S5.

### Analysis of intraspecies sequence-level diversity in core genomes

For each species and core gene, the frequency of each observed coding variant was counted and the Shannon entropy of this variant count distribution plus a dummy variant with frequency 1 (in order to distinguish genes with similar variant relative frequencies but different raw counts) was computed, referred to as the “coding allelic entropy” of the gene for that species. Analogous 5′ intergenic and 3′ intergenic allelic entropies were also computed per gene based on distributions of their respective variant types. Core genome-wide Spearman correlations were computed between these three allelic entropies for each pair of variant types, for each species.

To control for the effect of gene length on the number of unique coding variants and thus on coding allelic entropy, quantile regression was used to determine the 5 and 95% coding allelic entropy percentiles as a quadratic function of gene length [24], using Python package statsmodels [52]; the quadratic functional form was chosen as it was the simplest form that closely tracked the rolling window 5 and 95% percentiles (Fig. S8). Functional enrichment among the most conserved and diverse core genes (determined by the 5 and 95% quantile regression percentiles) was computed similarly as for the frequency group enrichment tests, computing LORs and applying Fisher’s exact tests for each COG functional category. A total of 2 groups (top/bottom 5%) × 20 COGs × 12 species = 480 tests were conducted, and significance was determined based on FWER < 0.5 under Bonferroni correction, or *p*-value < 1*10^− 4^. Similar enrichment tests were conducted for the top/bottom 5% of core genes ranked by either intergenic allelic entropy measure, using regular 5%/95% quantiles not based on quantile regression since intergenic features were fixed-length. All LORs and *p*-values are available in Dataset S4.

### Analysis of sequence-level diversity in MLST genes

DNA sequences for genes involved in PubMLST typing schemes were downloaded through the mlst tool v2.18.0 (https://github.com/tseemann/mlst). Each sequence was translated to an amino acid sequence in the frame with the fewest number of intermediate stop codons. Within each species, pangenome coding variants were mapped to translated PubMLST variants if they contained the exact sequence of the translated PubMLST variant, to yield variant-variant mappings. Pangenome genes were then mapped to PubMLST genes based on which pangenome gene had the maximum number of variant-variant mappings to a given PubMLST gene. The coding allelic entropies of the pangenome genes mapped to PubMLST genes were reported, as percentiles relative to the coding allelic entropies of all core genes for the species.

### Analysis of sequence variation positional distribution with respect to domains

The 168 genes previously identified to be in all 12 core genomes were filtered for those with rich domain annotations. Starting with *E. coli* amino acid sequences, for each gene: 1) a multiple sequence alignment (MSA) was computed for all observed coding variants using MAFFT [25], 2) the consensus sequence was annotated for domains with InterProScan [26], 3) domains with the same InterPro accession ID were merged, 4) and domains longer than 80% of the full protein length were filtered out. This analysis yielded 76 genes with at least three domain annotations, and the amino acid sequences related to these genes for all 12 species were similarly analyzed for a total of 912 species-gene pairs annotated. Gene name annotations were assigned based on earlier eggNOG-mapper annotations of the most common sequence variant.

To quantify domain sequence variation, the entropy at each position of each species-gene MSA was computed, weighted by the relative abundance of each sequence variant. For each domain, the mean entropy across all MSA positions spanned by the domain was computed, and the entropy’s percentile was computed against the mean entropies of all subsequences of the same length within the MSA, yielding entropy percentiles for each species-gene-domain combination. To determine domains consistently variable or conserved across multiple species, the mean entropy percentile for each gene-domain pair was computed across the 12 species. *P*-values of mean entropy percentiles were determined against an empirically constructed distribution for the mean of 12 independent, identically distributed uniform distributions using 1,000,000 random samples (Bates distribution, based on a null hypothesis of percentiles being uniformly distributed). Significance was determined based on Benjamini-Hochberg correction (FDR < 0.05, 443 tests). All domain entropy percentiles, *p*-values, and significance calls are available in Dataset S6.

Domains related to aminoacyl-tRNA synthetases (AARSs) were identified based on gene name annotations. Functional categories (editing, anticodon binding, tRNA binding, non-editing catalytic) were assigned based on InterPro text annotations of each domain. Between domains overlapping by more than 95%, only the domain with a functional annotation (rather than structural) and/or more descriptive InterPro annotation was shown as representative. A summary of domain functional assignments, evidence, and overlap filtering is available in Dataset S6.

## Supplementary Information

**Additional file 1: Figure S1.** Phylogenetic tree of selected species and MLST subtype distributions. a) Phylogenetic tree constructed for representative genomes of each species using PATRIC’s Codon Tree service. Genomes are labeled by their name and PATRIC Genome ID. b) Distribution of MLST subtypes for each species’ genome collection. The relative abundance of the top 5 MLST subtypes, all other subtypes, and untyped genomes are shown per species. **Figure S2.** Evaluation of accuracy of Heaps’ Law at predicting pangenome size, with or without controlling from MLST. a) Example fit of Heaps’ Law to first half of genomes (unbalanced) or MLSTs (MLST balanced) and extrapolation to second half to evaluate pangenome size projection. b-c) Median mean absolute error (MAE) across Heaps’ Law fits for 100 random genome orderings, with or without MLST balancing, for each species in the fitting and extrapolation regions. Dotted lines indicate equal performance between the two methods. **Figure S3.** Gene frequency distributions by species. **Figure S4.** Fitted cumulative gene frequency distributions and corresponding core and unique gene frequency thresholds, by species. Observed distributions (solid blue), fitted functions (dashed orange), and the R^2^ and mean absolute errors (MAE) of the fits are shown. Fitted inflection points (black dot, dashed gray) and frequency thresholds corresponding to core and unique genes (dashed black) are also shown. **Figure S5.** COG functional group enrichment in the core, accessory, and unique genomes of 12 species. Heatmaps are colored by the log2 odds ratio (LOR) between each COG and the a) core, b) accessory, c) unique genome of each species. COGs are sorted by mean LOR across all species. LOR color scales are symmetric and identical for all plots; four values are outside of the color range: F x *E. cloacae* (LOR = − 7.5), Q x *C. coli* (LOR = − 6.0), and K x *C. coli* (LOR = − 6.9) for accessory genomes; F x *E. faecium* (LOR = − 5.3) for unique genomes. Starred cells correspond to statistically significant enrichments under Fisher’s Exact test with FWER < 0.05 under Bonferroni correction (*p* < 7*10^− 5^, 720 tests). **Figure S6.** Top 10 GO terms by enrichment in the core, accessory, and unique genomes of 12 species. Heatmaps are colored by the log2 odds ratio (LOR) between each GO term and the a) core, b) accessory, or c) unique genome of each species. GO terms are sorted by mean LOR across all species. LOR color scales are identical for all plots. Starred cells correspond to statistically significant enrichments under Fisher’s Exact test with FWER < 0.05 under Bonferroni correction (*p* < 3*10^− 6^, 14,904 tests). **Figure S7.** Quantile regression between coding allelic entropy and gene length among core genes, by species. Dotted lines show quantile regressions for the 5 and 95% coding allelic entropy percentiles as a quadratic function of gene length. Red and blue dots are the most diverse and most conserved core genes, respectively, as defined by these regressions. **Figure S8.** Rolling window percentiles versus quantile regression between coding allelic entropy and gene length among core genes, by species. Dotted lines show quantile regressions for the 5 and 95% coding allelic entropy percentiles as a quadratic function of gene length. Orange lines show rolling 5 and 95% percentiles using windows of 50 genes. **Figure S9.** Coding allelic entropies of genes used in MLST typing schemes, as percentiles among all core genes of the corresponding species. For *A. baumannii*, the MLST gene *gpi* was mapped to two pangenome gene clusters denoted gpi-1 and gpi-2, both of which include *gpi* variants defined in the *A. baumannii* MLST typing scheme. **Figure S10.** Domains with significant mutation depletion across multiple species. Species-specific mutation depletion for gene-domain pairs with significant multispecies mutation depletion (Bootstrap test, FDR < 0.05, Benjamini-Hochberg correction). Domains related to aminoacyl-tRNA synthetases are labeled purple. White cells correspond to domains that could not be annotated within the species’ consensus sequence of the parent protein. **Table S1.** Genome counts, abbreviations, and taxon IDs for species examined. **Table S2.** Heaps’ Law parameter estimates, fitted by either randomly shuffling all genomes “by genome” or one genome per MLST “by MLST.” Means and standard deviations from 100 iterations are shown for each species, parameter, and method. Species are sorted by Heaps’ Law alpha, estimated using the MLST method. **Table S3.** Evaluating accuracy of Heaps’ Law fits, based on either randomly shuffling all genomes “by genome” or one genome per MLST “by MLST.” Heaps’ Law was fit to the first half of genomes in pangenome size curves (“fitting region”) generated by either method and accuracy was evaluated against the second half (“extrapolation region”). The mean absolute error (MAE) for each region was computed, and the median MAE across 100 iterations is shown, as well as relative error between the MLST vs genome methods. Species are sorted by relative median MAE in the extrapolation region. **Table S4.** Gene frequency cutoffs and gene counts for the core, accessory, and unique genomes of 12 species. **Table S5.** Correlations between three types of intraspecies sequence diversity for core genes. Variant types are coding (protein sequences), 5′ intergenic (5′ IG, 300 nt upstream and adjacent to the start codon), and 3′ intergenic (3′ IG, 300 nt downstream and adjacent to the stop codon). **Dataset S1.** PATRIC genome IDs for all genomes used. **Dataset S2.** MLST annotations generated with https://github.com/tseemann/mlst for all genomes. **Dataset S3.** Summary of double power function fits to cumulative gene frequency distributions and derived thresholds for classifying genes as core, accessory, or unique. Includes for each species the minimum frequency to classify a gene as core, maximum frequency to classify a gene as unique, sizes of the core/accessory/unique genomes, R^2^ and MAE of the fit, and the five fitted parameters. **Dataset S4.** Log odd ratios and Fisher’s exact test *p*-values for enrichment between gene functional groups (COGs, GO terms) and various gene categories (core, accessory, unique, highest sequence diversity, lowest sequence diversity) within each species. Contains raw data for heatmaps and boxplots in Fig. 3c, Fig. 5b, Fig. S5, and Fig. S6. **Dataset S5.** Predicted gene names, COG functional categories, and TraDIS *E. coli* essentiality predictions from Goodall et.al. 2018 for the 168 genes observed in the core genome of all 12 species. **Dataset S6.** Domain mutation enrichment analysis. For each gene-domain pair, includes the estimated mutation enrichment as domain entropy percentile (species-specific and species-wide averages), Bootstrap test p-values, domain InterPro accession IDs, and domain descriptions. Also includes assignment of functional categories to AARS-related domains.

## Data Availability

All genome sequences used in this study are publicly available on the PATRIC database (https://www.patricbrc.org/). Genome IDs for sequences used are available in Dataset S1.
