# Supplementary material for: Comparative pangenomics: analysis of 12 microbial pathogen pangenomes reveals conserved global structures of genetic and functional diversity
Source: BMC Genomics. 2022 Jan 4;23:7. doi: 10.1186/s12864-021-08223-8 (PMC8725406; doi:10.1186/s12864-021-08223-8)
Supplement: Supplementary file 1 — Additional file 1: Figure S1. Phylogenetic tree of selected species and MLST subtype distributions. a) Phylogenetic tree constructed for representative genomes of each species using PATRIC’s Codon Tree service. Genomes are labeled by their name and PATRIC Genome ID. b) Distribution of MLST subtypes for each species’ genome collection. The relative abundance of the top 5 MLST subtypes, all other subtypes, and untyped genomes are shown per species. Figure S2. Evaluation of accuracy of Heaps’ Law at predicting pangenome size, with or without controlling from MLST. a) Example fit of Heaps’ Law to first half of genomes (unbalanced) or MLSTs (MLST balanced) and extrapolation to second half to evaluate pangenome size projection. b-c) Median mean absolute error (MAE) across Heaps’ Law fits for 100 random genome orderings, with or without MLST balancing, for each species in the fitting and extrapolation regions. Dotted lines indicate equal performance between the two methods. Figure S3. Gene frequency distributions by species. Figure S4. Fitted cumulative gene frequency distributions and corresponding core and unique gene frequency thresholds, by species. Observed distributions (solid blue), fitted functions (dashed orange), and the R2 and mean absolute errors (MAE) of the fits are shown. Fitted inflection points (black dot, dashed gray) and frequency thresholds corresponding to core and unique genes (dashed black) are also shown. Figure S5. COG functional group enrichment in the core, accessory, and unique genomes of 12 species. Heatmaps are colored by the log2 odds ratio (LOR) between each COG and the a) core, b) accessory, c) unique genome of each species. COGs are sorted by mean LOR across all species. LOR color scales are symmetric and identical for all plots; four values are outside of the color range: F x E. cloacae (LOR = − 7.5), Q x C. coli (LOR = − 6.0), and K x C. coli (LOR = − 6.9) for accessory genomes; F x E. faecium (LOR = − 5.3) for unique genomes. Starred ce [file 12864_2021_8223_MOESM1_ESM.zip › FigS4.pdf]

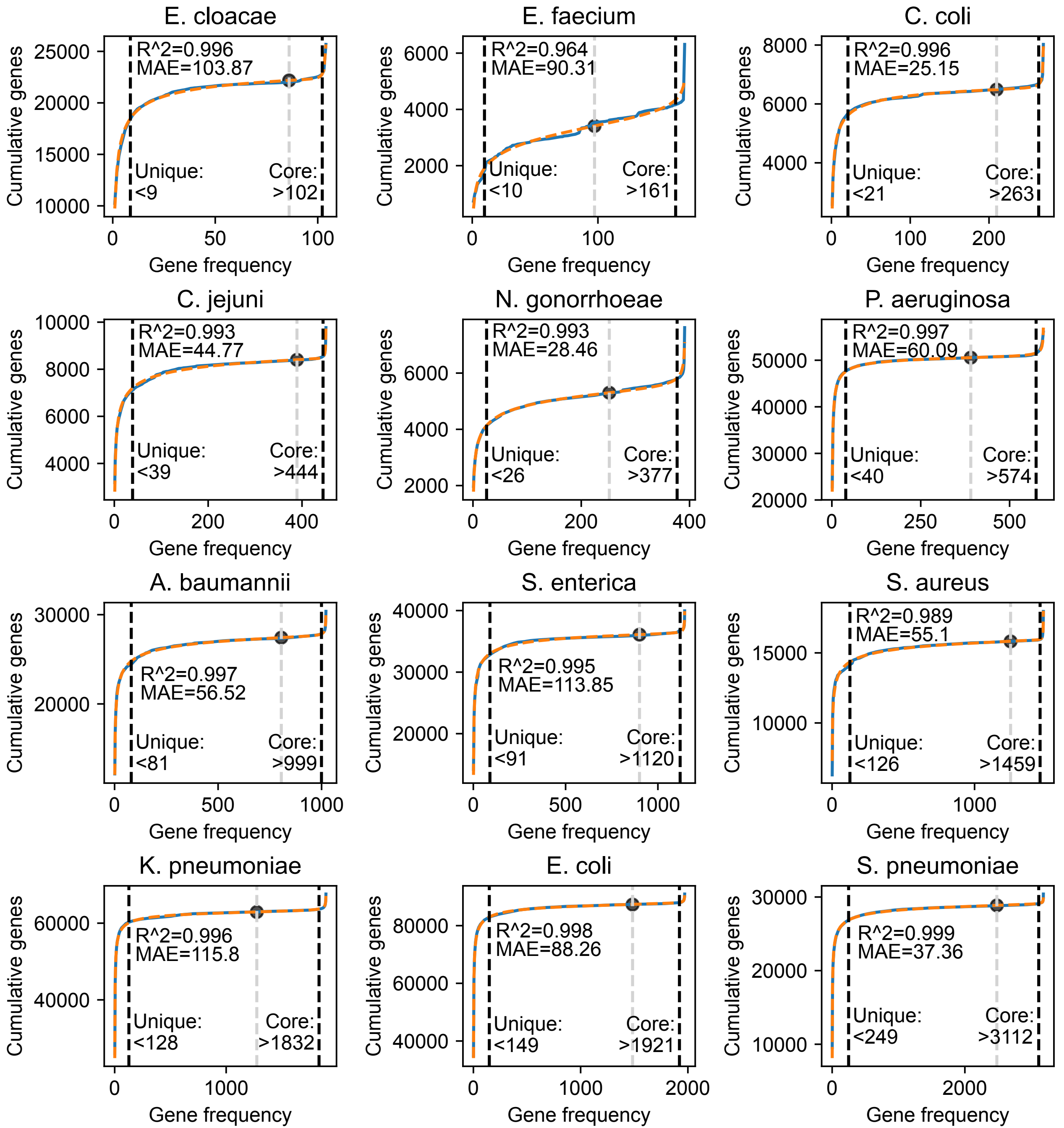

E. cloacae
R^2=0.996
MAE=103.87
Unique:
<9
Core:
>102
E. faecium
R^2=0.964
MAE=90.31
Unique:
<10
Core:
>161
C. coli
R^2=0.996
MAE=25.15
Unique:
<21
Core:
>263
C. jejuni
R^2=0.993
MAE=44.77
Unique:
<39
Core:
>444
N. gonorrhoeae
R^2=0.993
MAE=28.46
Unique:
<26
Core:
>377
P. aeruginosa
R^2=0.997
MAE=60.09
Unique:
<40
Core:
>574
A. baumannii
R^2=0.997
MAE=56.52
Unique:
<81
Core:
>999
S. enterica
R^2=0.995
MAE=113.85
Unique:
<91
Core:
>1120
S. aureus
R^2=0.989
MAE=55.1
Unique:
<126
Core:
>1459
K. pneumoniae
R^2=0.996
MAE=115.8
Unique:
<128
Core:
>1832
E. coli
R^2=0.998
MAE=88.26
Unique:
<149
Core:
>1921
S. pneumoniae
R^2=0.999
MAE=37.36
Unique:
<249
Core:
>3112
Cumulative genes
Gene frequency
